# Oral Chronic Mucositis in a Known Lichen Planus Pigmentosus Patient

**DOI:** 10.1155/2024/1975932

**Published:** 2024-06-14

**Authors:** Dalad Pinchaleaw, Pirawish Limlawan

**Affiliations:** ^1^ Residency Training Department of Oral Medicine Faculty of Dentistry Chulalongkorn University, Pathumwan, Bangkok 10330, Thailand; ^2^ Department of Oral Medicine Faculty of Dentistry Chulalongkorn University, Pathumwan, Bangkok 10330, Thailand

## Abstract

Oral manifestations of mucocutaneous inflammatory diseases such as lichenoid dermatoses often affect the patient's quality of life through the symptoms of pain and burning sensation. In this case report, a patient with known lichen planus pigmentosus (LPP), a variant of lichenoid dermatoses that rarely affect oral mucosa, presented with chronic pain in the oral cavity for 2 months. Her intraoral examination revealed multiple pseudomembranous ulcers with erythematous areas and white striae similar to erosive lichen planus. The histological assessment revealed chronic mucositis, while the direct immunofluorescent examination showed negative results, contradicting the diagnosis of both lichen planus and LPP. Thus, the diagnosis was given as chronic mucositis, and the topical steroid was given. After 6 weeks of steroid application, the lesion resolved. To the best of our knowledge, this is the first report of chronic mucositis in LPP patients. This report could raise the awareness of clinicians to carefully take history as the patient with LPP may suffer from chronic mucositis in the oral cavity as well.

## 1. Introduction

Lichen planus pigmentosus (LPP), a rare subvariant of lichenoid dermatoses, is a group of mucocutaneous inflammatory diseases of unknown etiology consisting of lichen planus (LP), which is the most common, and lichen planopilaris [1]. This idiopathic inflammatory condition of the skin is characterized by chronic-acquired dark brown to gray macular pigmentation at sun-exposed areas of the face and neck and sun-protected flexural skin in dark-skinned middle-aged patients which rarely involves oral mucosa. Although the etiology and pathogenesis are still unclear, it has been discovered that some of them are associated with the hepatitis C virus and other variants of LP. Affected patients may suffer from emotional stress due to the manifestation of the lesion in an aesthetic area and the chronic nature of the disease, which impact their quality of life [2].

This case report details the presentation of a 70-year-old female patient initially diagnosed with oral lichen planus (OLP), alongside LPP as her known underlying condition. However, an incisional biopsy yielded findings indicative of chronic mucositis instead. One week later, without any treatment, the lesions improved, and then, with the aid of a topical steroid supplement, the lesions eventually resolved.

## 2. Case Presentation

A 70-year-old female patient was referred to the Post-graduate of Oral Medicine Department, Faculty of Dentistry, Chulalongkorn University, with oral lesions on her tongue and both sides of the buccal mucosa with chronic pain and burning sensation rated by a numeric rating score of 3/10 for 2 months as her chief complaint, and there was no period of symptom improvement. The patient has lichen planus pigmentosus (LPP) (diagnosed by a skin biopsy a long time ago) and kidney disease. She does not take any medication but periodically follows up at the hospital every 2 weeks for LPP and 4 months for a kidney condition. The patient declined drug or food allergies. She took magnesium; beta-glucan; vitamin B1, 3, and 12; vitamin C; zinc; and fiber as supplements.

The last laboratory investigation was done 3 weeks before the visit which revealed a low red blood cell count, mean corpuscular hemoglobin concentration (MCHC), lymphocyte, high mean corpuscular volume (MCV), and neutrophil.

Other special tests revealed a nonreactive anti-HIV, negative LE cell, negative KOH staining for candida at the ventral of the tongue, and negative for both antinuclear antibody (ANA) and anticytoplasmic antibody. She detailed her symptoms and said that those pain and/or burning sensations also occurred spontaneously. She also could not take any hot or spicy foods, and she had to use a mild-tasting toothpaste that did not disturb her oral hygiene care.

**Figure 1:**
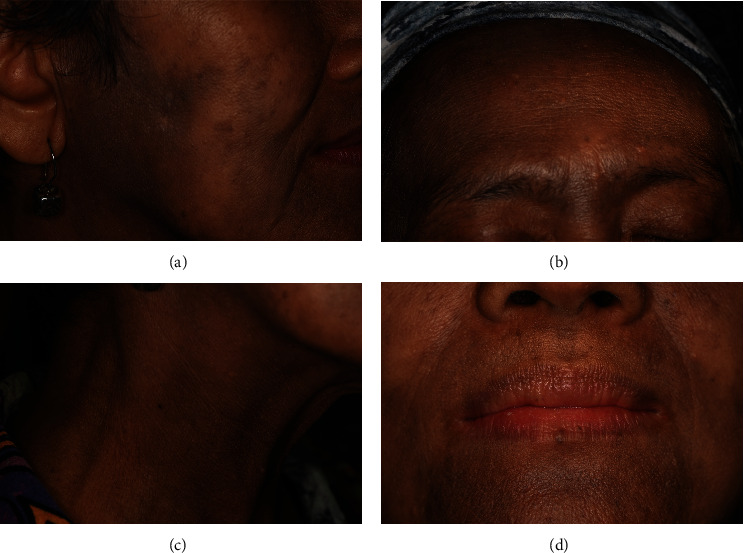
Extraoral examination demonstrated multiple brown macules scattered at the (a) cheek, (b) forehead, (c) neck, and (d) perioral.

The extraoral examination demonstrated multiple brown macules scattered around her face and neck area as a lesion of LPP (Figure 1).

**Figure 2:**
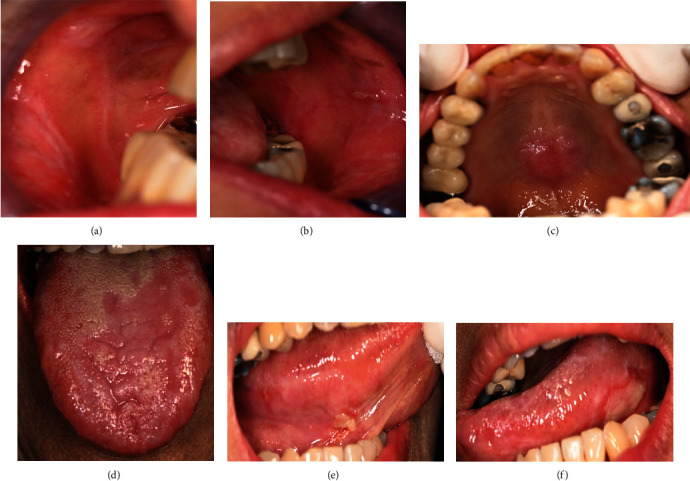
The first-visit intraoral examination revealed erythematous areas with white striae at the (a) right buccal mucosa, (b) left buccal mucosa, (c) palate, and (d) depapillated dorsum of the tongue. Pseudomembranous ulcer and erythematous areas with white striae at the (e) right lateral tongue and (f) left lateral tongue.

For intraoral examination (Figure 2), erythematous areas with white striae were found in several areas, including the lower lip, lower labial mucosa, labial gingiva of lower teeth extending to the mucobuccal fold, buccal gingiva of teeth 15-16 extending to the mucobuccal fold, mucobuccal fold of teeth 45-47, right buccal mucosa (Figure 2(a)), buccal gingiva of tooth 35, interdental papilla of tooth 35/36, left buccal mucosa (Figure 2(b)), mid-palate at the torus palatinus (Figure 2(c)), left side of the dorsum of the tongue (Figure 2(d)), ventral of the tongue (Figures 2(e) and 2(f)), and the floor of the mouth. Pseudomembranous ulcerations at the left buccal mucosa, size 3 × 4 mm^2^ (Figure 2(b)); the left lateral of the tongue, size 10 × 30 mm^2^ (Figure 2(f)); and the right ventral of the tongue in the area of teeth 43-45, size 5 × 8 mm^2^ (Figure 2(e)). The other finding was a white plaque which cannot be rubbed off at the right lateral of the tongue. There were some restorations in her mouth, such as tooth 37 as a full metal crown (FMC) and teeth 36, 14-17, 45, and 46 as a porcelain-fused-metal crown (PFM). There were amalgam fillings on teeth 35(OD) and 16(OM). There were also PFM bridges from teeth 23 to 26 and full metal bridges from teeth 45 to 47.

**Figure 3:**
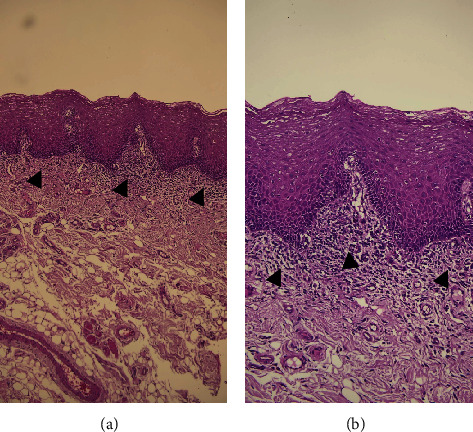
The microscopic examination reveals an oral mucosa surfaced by parakeratinized stratified squamous epithelium. Chronic inflammatory cell infiltration is observed within the superficial portion of the connective tissue (black arrow). Thick-walled vascular channels, adipose tissues, and muscle bundles are within the deeper portion of the connective tissue at the magnification of (a) 40x and (b) 100x.

After history-taking and clinical examination, the clinical impression was given as oral lichen planus (OLP). After explaining the process of treatment, two incisional biopsies were done at her left buccal mucosa for H&E staining and direct immunofluorescent examination (DIF). The patient received medication including paracetamol 500 mg 10 tablets for taking 1 tab as needed for pain, 2% sodium bicarbonate mouthwash for mouth rinsing, and glycerine solution to apply to her dry lip. One week later, the result of the incisional biopsy (Figure 3) revealed chronic mucositis similar to lichen planus or lichenoid mucositis but lacking the triad which are band-like infiltrates, irregular acanthosis, and vacuolation of basal cells known as “hallmark of LP” [3].

Because of the inconclusive biopsy result of H&E, DIF would be beneficial to give a more specific diagnosis [4]. The classic findings of OLP include shaggy staining of antifibrinogen with weak anti-C3 staining in basement membrane zone [3, 4]. However, in this case, DIF had all negative findings (Supplementary Figure 1). Thus, the final diagnosis was chronic mucositis.

**Figure 4:**
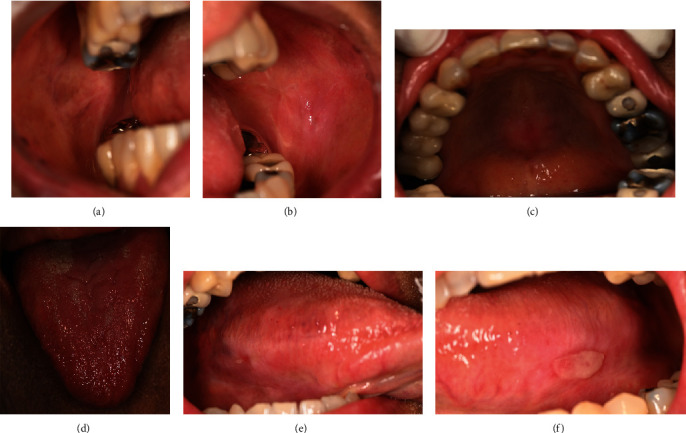
The second-visit intraoral examination revealed mild erythematous areas with white striae at the (a) right buccal mucosa, (b) left buccal mucosa, and (c) palate. (d) Depapillated dorsum of the tongue. Pseudomembranous ulcer at the (e) right lateral tongue and (f) left lateral tongue.

On the second visit (2 weeks later), the patient reported resolving symptoms with the left buccal mucosa as the most affected area. Clinical examination also showed resolving signs, described as a mild erythematous area with white striae at the left and right buccal mucosa (Figures 4(a) and 4(b)); mid-palate (torus palatinus) (Figure 4(c)); and left, right, and depapillated tongue with the erythematous area (Figure 4(d)). Pseudomembranous ulcers still persisted at the left and right lateral of the tongue (Figures 4(e) and 4(f)). Topical steroids including 0.1% fluocinolone acetonide + clotrimazole gel (Fluocogel) and 0.1% triamcinolone mouthwash (300 ml) were given to be applied to the lesion three times a day. The patient also continued using 2% sodium bicarbonate mouthwash and a glycerine solution.

**Figure 5:**
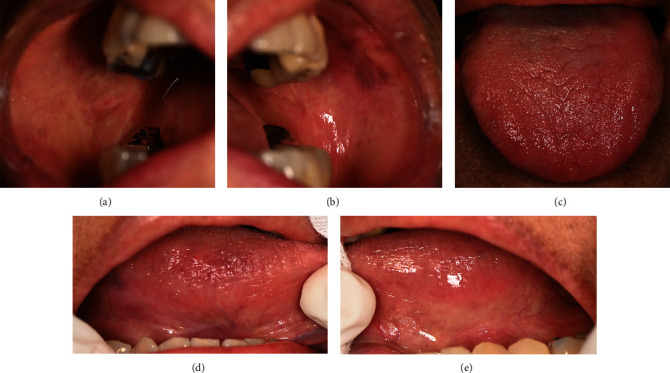
The third-visit intraoral examination revealed mild white striae and hyperpigmentation at the (a) right buccal mucosa and (b) left buccal mucosa. (c) Mildly depapillated dorsum of the tongue. Normal mucosa at the (d) right lateral tongue and (e) left lateral tongue.

On the third visit (2 weeks later), the patient reported no pain at any site, only a mild irritation at the right lateral of the tongue. She applied the medications as instructed. Clinical examination revealed mild white striae at the right buccal mucosa (Figure 5(a)), white striae at the mucobuccal fold area of teeth 36-37 extending to the left buccal mucosa (Figure 5(b)), mucobuccal fold of teeth 44-47, hyperpigmentation at the left and right buccal mucosa, and a mildly depapillated dorsal tongue (Figure 5(c)). The ulcers on both sides of the lateral tongue resolved (Figures 5(d) and 5(e)). The lesion on the palate was also completely resolved. The prescription was given the same as the previous visit, except for the reduction of the frequency of Fluocogel and 0.1% triamcinolone mouthwash from 3 to 2 times a day.

**Figure 6:**
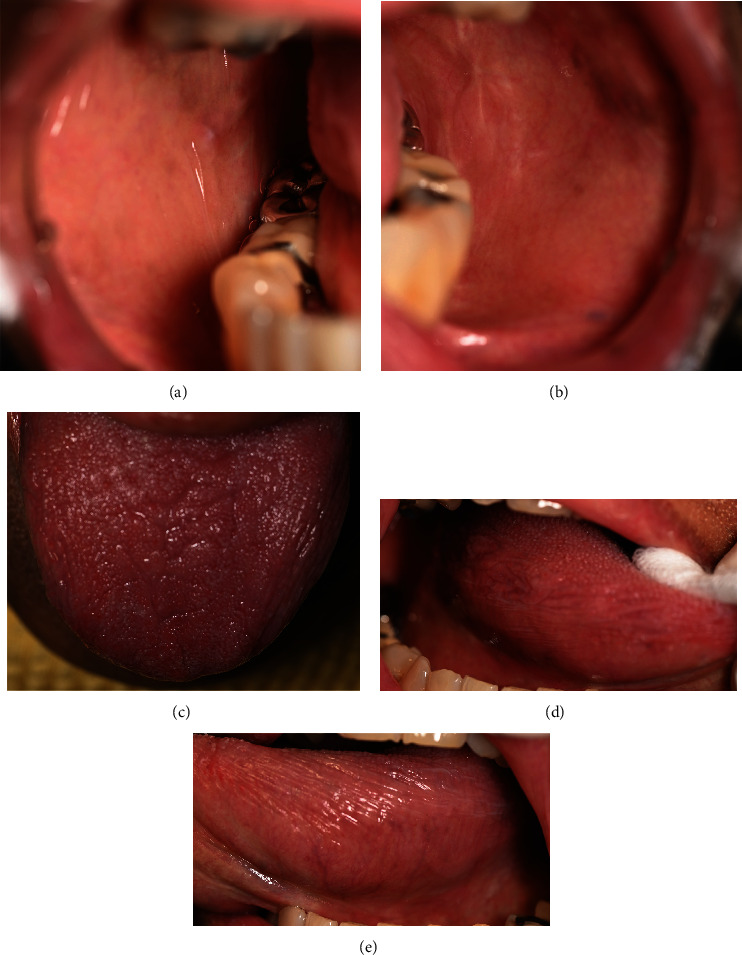
The fourth-visit intraoral examination revealed mild white striae and hyperpigmentation at the (a) right buccal mucosa and (b) left buccal mucosa. (c) Mildly depapillated dorsum of the tongue. Normal mucosa at the (d) right lateral tongue and (e) left lateral tongue.

**Figure 7:**
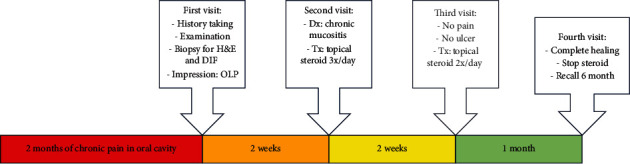
Timeline of the case from the beginning to the end. OLP = oral liche planus; H&E = hematoxylin and eosin; DIF = direct immunofluorescent; Dx = diagnosis; Tx = treatment.

On the last visit (1 month later), the patient came with only a mild burning sensation at the tongue and no pain in the other oral mucosa area. Clinical examination revealed a mild white line and postinflammatory hyperpigmentation at the left and right buccal mucosa with a mildly depapillated area at the dorsum of the tongue (Figure 6). We considered that the lesion had resolved and appointed the patient for a regular recall at 6 months. The patient was informed to revisit if the lesions recur before regular check-ups. To bring closure to the case, it took four visits over a span of two months to effectively address and resolve the illness, as outlined in Figure 7.

## 3. Discussion

Lichen planus pigmentosus (LPP) predominantly affects patients with darker skin (phototypes III to VI, with the highest prevalence in phototypes IV). The disease can be seen in India, Latin America, Asia, and Africa. However, it is rarely seen in Caucasians. The lesion occurs between the 3^rd^ and 5^th^ decades of life, with a low prevalence in childhood. LPP is more common in females [2].

Although its etiology remains unclear, some research suggested that topical use and consumption of photosensitizer (allyl-thiocyanate), amla oil, henna, hair dye, cold cream, and environmental pollution can be a trigger factor in India [5, 6]. Moreover, research on Kuwait patients also found hepatitis C virus infection as an exacerbating factor [7].

LPP lesions appear as a symmetrical distribution of dark brown to gray or gray-blue, round, oval, or irregular macules with ill-defined borders. The lesion mainly affects the face, especially the temporal and preauricular areas and all sides of the neck. The arms are affected more than the legs, and the lesion also involves flexural areas such as the axillae, inguinal creases, and inframammary folds [8]. Only a few studies reported that LPP affects the oral mucosa, and the lesion presents as pigmentation and white patches over bilateral buccal mucosae with a burning sensation, and none of the cases reveal pseudomembranous ulceration [9–11]. On the skin, mild itching was reported in 27-62% of the cases but eventually ceased with or without treatment. A burning sensation was also reported [5, 6]. LPP can impact the patient's quality of life due to its chronic and progressive remissions and exacerbations from 6 months to 3 years [12]. Previous studies reported that LPP can occur with lichen planus (LP), as active or resolving LP lesions can be found in patients with LPP [5, 13]. LPP also coexists with other variants of LP such as lichen planopilaris [14] and oral lichen planus (OLP) [15–17]. One study showed that LPP also correlates with autoimmune diseases such as Sjogren's syndrome, Crohn's disease, and rheumatoid arthritis [18].

In the present case, the patient is of Indian descent and presented with chronic pain in the oral mucosa for 2 months with known LPP. Her oral lesion resembles an OLP. We wondered whether it was an oral manifestation of LPP or concomitant OLP, and we biopsied for a final diagnosis. Previous studies reported the histopathology of oral LPP as hyperkeratosis with thickening of the granular cell layer, sawtooth-shaped rete pegs, a dense subepithelial band of chronic inflammatory cell infiltrate in lamina propria, and melanin incontinence [9–11]. However, the histopathology of this patient revealed mucosal inflammation without hallmarks of OLP which are band-like lymphocyte infiltration, acanthosis, and basal cell degeneration [3]. DIF was also done to aid in the diagnosis. DIF is positive in 37-97% of OLP cases, while in LPP, it is rarely positive (7-16%) [2]. Classic findings of OLP include shaggy staining of antifibrinogen with weak anti-C3 staining in the basement membrane zone [3]. In this case, DIF was negative, more likely to be LPP; however, the H&E result was inconclusive.

Due to the diagnosis process of OLP which involves a clinician's evaluation of the patient's clinical presentation and histopathologic findings by a pathologist called “clinicopathologic correlation,” there are some ambiguous cases that cannot define a final diagnosis as OLP in a biopsy procedure [19]. Chronic mucositis is one of the descriptive diagnoses of a histopathology condition that lacks some histopathologic features of OLP [4]. Thus, the final diagnosis in this case was chronic mucositis, and topical steroids were administered. After the treatment, the patient reported a better quality of life due to the absence of ulcers and a burning sensation. The patient also provided informed consent for the publication of this case report.

## 4. Conclusion

Up to date, there has been no report of an association between LPP and chronic mucositis condition. In this case, the patient suffers from chronic mucositis for 2 months before seeking treatment. However, after the diagnosis was made, the patient responded well to the topical steroid treatment within 6 weeks. Based on this case report, it is advisable to conduct a thorough history-taking and oral examination of patients with LPP, as they may also suffer from chronic mucositis.

## Data Availability

The data of this study are available from the corresponding author upon reasonable request.

## Supplementary Materials

Supplementary Materials Supplementary Figure 1: direct immunofluorescence study of the patient.
