# Supplementary figures and images for: Oral Chronic Mucositis in a Known Lichen Planus Pigmentosus Patient
Source: Case Rep Dent. 2024 Jun 14;2024:1975932. doi: 10.1155/2024/1975932 (PMC11192598; doi:10.1155/2024/1975932)

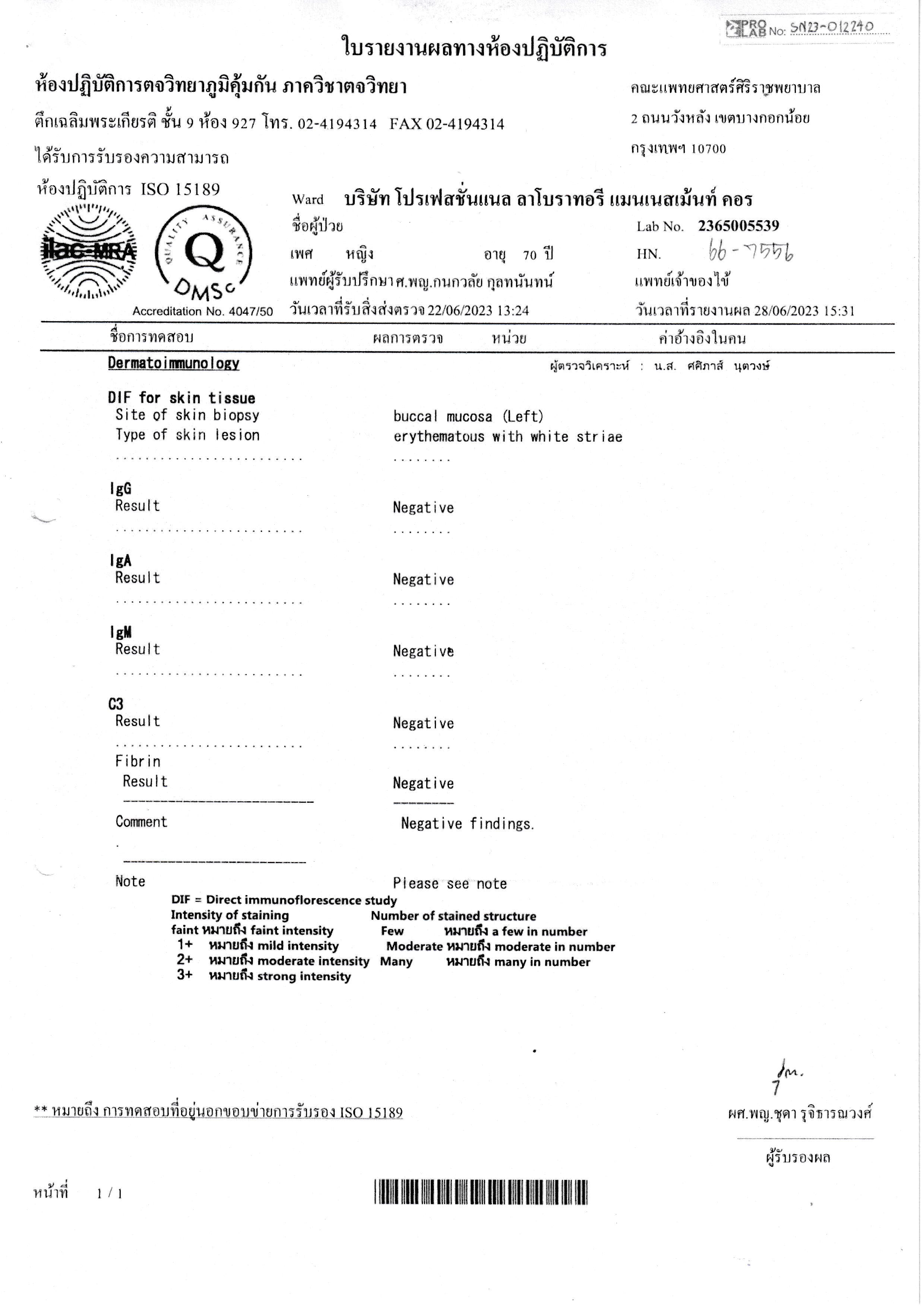

Supplement: Supplementary Materials — Supplementary Figure 1: direct immunofluorescence study of the patient. [file 1975932.f1.png]
